# Association between Free Sugars Intake and Risk of Metabolic Syndrome in Chinese Adults: Results from the China Health and Nutrition Survey, 2000–2018

**DOI:** 10.3390/nu14245385

**Published:** 2022-12-19

**Authors:** Feng Pan, Zhihong Wang, Huijun Wang, Chang Su, Jiguo Zhang, Wenwen Du, Xiaofang Jia, Liusen Wang, Hongru Jiang, Weiyi Li, Bing Zhang, Gangqiang Ding

**Affiliations:** National Institute for Nutrition and Health, Chinese Center for Disease Control and Prevention, Beijing 100050, China

**Keywords:** free sugars intake, metabolic syndrome, Chinese adults

## Abstract

The association of free sugars intake with metabolic syndrome (MetS) is controversial. This study aimed to examine the association between free sugars intake and risk of MetS in Chinese adults. The data were from seven waves of the China Health and Nutrition Survey (2000–2018), a longitudinal and open prospective cohort study. Dietary intake was calculated based on the data collected by consecutive 3-day 24-h dietary recalls combined with household weighing for foods or condiments. Cox proportion hazard regressions and restricted cubic spline (RCS) were performed to explore the associations between free sugars intake and the risk of MetS. The present study selected 12,048 adults aged 18 years and above. During a median (IQR) follow-up of 9.0 (7.0, 15.0) years with 131,844.0 person-years, 3970 (32.95%) MetS occurred. After adjusting for all potential confounders, compared to adults with free sugars intake of <5 g/day group, adults with free sugars intake of 5–20 g/day were associated with a higher risk of MetS (HR, 1.094; 95% CI, 1.009–1.186). No significant association was observed between free sugars intake of >20 g/day and the risk of MetS (HR, 1.011; 95% CI, 0.800–1.277). There is an urgent need to pay attention to the intake of free sugars and comprehensive measures such as the improvements in the processing of sugary foods, and sugar composition should be included on food nutrition labels to control intake of free sugars in China.

## 1. Introduction

Metabolic syndrome (MetS) is defined as a cluster of metabolic alterations including abdominal obesity, abnormal high-density lipoprotein cholesterol (HDL-C), elevated triglyceride (TG), high fasting blood glucose, and hypertension [1]. MetS has become a major public health concern, which can contribute to a higher risk of type 2 diabetes (T2D), cardiovascular disease (CVD), stroke, coronary heart disease, and all-cause mortality [2]. The study of the National Health and Nutrition Examination Survey (NHANES, 2011–2016) has shown that the MetS prevalence was 34.7% among adults in the United States [3]. According to data from China Nutrition and Health Surveillance (2015–2017), nearly a third (31.1%) of adults aged 20 and above had MetS in China [4]. Several studies suggest that food and nutrients are thought to play an important role in the development of MetS. “Free sugars” refer to monosaccharides and disaccharides which are added to foods and beverages by the manufacturer, cook, or consumer, and sugars naturally present in syrups, honey, fruit juices, and fruit juice concentrates, according to the World Health Organization (WHO). Free sugars may promote a positive energy balance and increase overall energy intake [5]. More and more epidemiologic studies have proven that intake of free sugars—particularly in the form of sugar-sweetened beverages (SSBs)—contribute to weight gain, MetS, and increased risk of noncommunicable diseases (NCDs) [6,7,8,9,10]. Nevertheless, the association of free sugars intake with MetS remains controversial [11]. Studies have found that sugar consumption can increase the risk of MetS that associates with excess ectopic fat accumulation [12]. Okuda et al. showed that there are no significant associations between high added sugars intake and MetS among Japanese adolescents [13]. Data from some RCTs and meta-analyses showed that there are no significant linkages between various adverse metabolic health effects and sugar consumption at normal levels within diets [14]. Given the sugary foods intake and increasing prevalence of MetS of Chinese residents, it is especially important to determine whether free sugars intake is associated with the risk of MetS. Therefore, the present study investigated the association between the intake of free sugars and the risk of MetS in Chinese adults aged 18 and above from the China Health and Nutrition Survey (CHNS 2000–2018) and put forward specific suggestions and strategies to reduce sugar intake and prevent MetS.

## 2. Materials and Methods

### 2.1. Study Population

All data used in current study were derived from the CHNS, which was a prospective and longitudinal cohort survey in China. A multistage, random cluster process was used to draw the sample in each province to ensure that urban and rural areas were represented. The CHNS aimed to develop a longitudinal survey that would understand how demographic and social economic changes in China affected health and health behaviors. The CHNS was designed to provide a representation of rural and urban areas varying in economy, geography, and health indicators with examining household-level and individual-level demographic factors, diet, health behavior, physical activity, and behavior changes. The background, aims, design, and methods of the survey have been described in detail elsewhere [15,16].

Our analysis used the seven waves of survey data between 2000 and 2018. All participants aged 18 and above who had complete data on dietary, socioeconomic, demographic, anthropometric, and other lifestyle factors were included. We excluded those that had MetS at baseline; those with implausible energy intakes (men: <800 kcal per day or >6000 kcal; women: <600 kcal or >4000 kcal); those with missing weight, height, serum lipids, blood pressure, blood glucose; those only in one wave before MetS was diagnosed; and those pregnant or lactating women during the survey. A total of 12,048 adults (5812 men and 6236 women) were included in the current analysis.

The survey was approved by the Institutional Review Committees of the University of North Carolina at Chapel Hill (UNC-CH) and the National Institute for Nutrition and Health, Chinese Center for Disease Control and Prevention (No. 201524). Written informed consent was obtained from all subjects.

### 2.2. Definition of Metabolic Syndrome

According to the National Cholesterol Education Program Adult Treatment Panel III (NCEP ATP III) criteria, MetS was defined if three out of five of the following components are present: (1) waist circumference ≥90 cm for men and ≥80 cm for women; (2) fasting triglycerides (TG) ≥ 150 mg/dL or specific treatment for lipid abnormality; (3) high-density lipoprotein cholesterol (HDL-C) < 1.0 mmol/L in men and 1.3 mmol/L in women; (4) diastolic blood pressure ≥ 85 mmHg or systolic blood pressure ≥ 130 mmHg, or treatment of previously diagnosed hypertension; (5) Fasting plasma glucose ≥ 6.0 mmol/L or diagnosed type 2 diabetes previously [17].

### 2.3. Measurement of Indicators

At least two trained health workers or nurses calibrated the instrument before taking physical measurements. Waist circumference, weight, and height were measured using a non-retractable material flexible ruler, electronic weight scale, SECA206 altimeter. Blood pressure was measured by a standard mercury sphygmomanometer (SBP and DBP were determined according to Korotkoff sound). After at least five minutes of rest in a quiet room, the participants were in a seated position and with the bladder emptied, and three consecutive standard measurements were taken for each participant to obtain the average value. TG and HDL-C were measured using cholesterol oxidase-phenol and an amino phenazone method. Fasting plasma glucose was measured by a Roche 702 instrument using the hexokinase method.

### 2.4. Assessment of Dietary Free Sugars

Dietary data were collected by consecutive 3-day 24-h dietary recalls (2 weekdays and 1 weekend) for each individual and seasonings in the household inventory were weighed. Quantities and types of all food consumed at home and away from home during the preceding 24 h were collected by trained investigators. Food consumption at the household level, such as oil, salt, sugar, and other condiments, was calculated by the ratio of each participant’s energy intake to the energy intake of the whole family and times of eating at home.

For the free sugars content in food, we used High performance Liquid Chromatography (HPLC) to determine the content of different kinds of food including SSBs, bakery products, candies, and chocolate. For some foods such as sauces and honey, the free sugars content was used from the China Food Composition Table (2004/2009) [18,19] and Food Nutrition Facts.

### 2.5. Definition of Covariates

Covariates including gender, age, education, geographic region, per capita annual family income, alcohol drinking history, smoking history, and physical activity were collected by trained interviewers through standard questionnaires. All activities including leisure time, household chores, occupational, and transportation activities in average hours per week were reported by participants, and then were converted into a metabolic equivalent-hours/week (MET-h/week) based on the American College of Sports Medicine Association’s recommended standard [20]. The individual annual income, physical activity, and energy intake were categorized into tertiles (low, medium, and high) in the analyses.

### 2.6. Statistical Analysis

Chi-square test for categorical variables were used to examine the differences of sociodemographic characteristics and lifestyles across different daily free sugars intake levels. The association between free sugars intake and the risk of MetS was explored by a multivariable Cox of proportional hazards model to estimate the hazard ratios (HRs) and 95% CIs. We constructed three sequential models. Model 1 adjusted for baseline age, gender, educational level, place of residence, region of residence, educational level, individual annual income, drinking history, and smoking history. Model 2 was further adjusted for physical activity based on Model 1. Model 3 was further adjusted for total energy intake based on Model 2.

To assess the nonlinear association between free sugars intake and the risk of MetS, restricted cubic spine (RCS) regressions were performed with the same covariates adjusted in the primary analysis among overall participants and subgroups for men and women.

All statistical analyses were performed using SAS version 9.4 software (SAS Institute Inc., Cary, NC, USA) and R version 4.1.0 (R Development Core Team, Vienna, Austria). All statistical tests were two-tailed and considered significant at *p* < 0.05.

## 3. Results

### 3.1. Basic Characteristics of Participants

A total of 12,048 participants were included in our study; 5812 (48.2%) were men and 6236 (51.8%) were women. Of the participants, 9056 (75.2%) consumed free sugars < 5 g/day, 2645 (22.0%) consumed 5–20 g/day, and 347 (2.9%) consumed >20 g/day. The median (IQR) age at baseline was 43.9 (33.9, 54.4) years. Among all the subgroups, the proportion was higher among participants who consumed free sugars < 5 g/day than those who consumed 5–20 g/day or >20 g/day. The proportion of participants that had a senior high education level, from urban areas, and had high income was almost about one third, respectively (Table 1).

For men, the proportion was higher among participants who had drinking history or smoking history with free sugars intake of <5 g/day and 5–20 g/day, respectively. The proportion was lower among participants who had low energy intake with free sugars intake of <5 g/day and 5–20 g/day, respectively. For women, the opposite is true for men (Table 2 and Table 3).

### 3.2. Association between Free Sugars Intake and the Risk of MetS

Table 2 shows the longitudinal association between free sugars intake and the risk of MetS in overall participants and stratified by gender. During a median (IQR) follow-up of 9.0 (7.0, 15.0) years with 131,844.0 person-years, 3970 (32.95%) MetS occurred. After adjusting for potential confounders in each model, compared to adults with free sugars intake <5 g/day group, adults with free sugars intake of 5–20 g/day were associated with a higher risk of MetS (HR, 1.094; 95% CI, 1.009–1.186). No significant association was observed between free sugars intake of >20 g/day and the risk of MetS (HR, 1.011; 95% CI, 0.800–1.277).

The results were different between gender-stratified analyses. No association was observed among men with free sugars intake of 5–20 g/day (HR, 1.077; 95% CI, 0.957–1.213) and >20 g/day (HR, 1.073; 95% CI, 0.782–1.473) compared to <5 g/day free sugars intake group. Women with free sugars intake of 5–20 g/day were associated with a higher risk of MetS (HR, 1.122; 95% CI, 1.003–1.255) (Table 4).

### 3.3. Nonlinear Associations between Free Sugars Intake and the Risk of MetS

**Figure 1:**
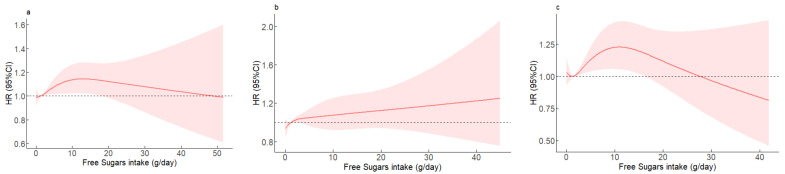
The relationships between free sugars intake and the risk of MetS during follow-up. The hazard ratio for MetS with the corresponding 95% confidence interval as a function of free sugars intake from Cox proportional hazard regression models adjusted for gender, age, educational level, place of residence, region of residence, individual annual income, drinking history, smoking history, physical activity, and total energy. (**a**) overall participants, *p* for nonlinear = 0.1623; (**b**) men, *p* for nonlinear = 0.4183; (**c**) women, *p* for nonlinear = 0.0374.

Estimated associations between free sugars intake and the risk of MetS are shown from nonlinear RCS models among the overall participants, men, and women (Figure 1). Among the overall participants, free sugars intake of 5–20 g/day was associated with a higher risk of MetS, whereas >20 g/day was not associated with MetS. Women with free sugars intake of 5–20 g/day were associated with a higher risk of MetS while no significant association was observed for men.

## 4. Discussion

Using CHNS 2000–2018, this study examined the association between free sugars intake and risk of MetS among Chinese adults. A significantly higher consumption of free sugars was found among adults who had senior high school or above education, lived in urban areas, had higher income, or had lower physical activity. We found that high consumption of free sugars was associated with an increased risk of MetS. Furthermore, free sugars intake of 5–20 g/day was positively associated with MetS in Chinese women, whereas a similar association was not observed in Chinese men.

The health effects of free sugars intake on people have been discussed for many years. More and more studies focus on the association between free sugars intake and chronic noncommunicable diseases such as overweight/obesity [21,22], diabetes [23], and cardiovascular diseases [6]. In a cross-sectional analysis of the NHANES (2005–2012), Rodríguez et al. found that, in the highest quintile of added sugar intake, the risk of MetS increased by 8.7 times in adolescents (POR = 8.7, 95% CI = 1.4–54.9) [24]. In the study of 7005 adults aged 40–69 years in Korea, the males had a significantly higher odds of MetS (OR = 1.332, 95% CI = 1.038–1.709) than those who with a lower ratio of energy from total sugar, but women did not [25]. Nevertheless, in our study, we found a positive association between free sugars intake and MetS in women, but not in men. It is unclear why men and women showed a different association between sugar intake and metabolic disease. It might be explained by dietary behavior differences between men and women that could alter the metabolic profile and level of hormones, which could affect metabolic disease.

There are several explanations and mechanisms for the association between sugar intake and cardiometabolic risk factors. Free sugars were provided mostly by SSBs and sweet products in the diet [26,27]. The energy can be absorbed rapidly with the form of free sugar or SSBs. Then, a positive energy balance happened and increased weight [28]. Moreover, some evidence suggests that free sugars (glucose, sucrose, and fructose) seem to interfere with the metabolism of essential fatty acids and decrease the formation of arachidonic acid, eicosapentaenoic acid, and docosahexaenoic acid, which could predispose to the development of metabolic syndrome [29].

In 2015, WHO recommended a reduced intake of free sugars throughout one’s whole life. Furthermore, it is recommended to reduce the intake of free sugars to <10% of total energy intake and a further reduction of the intake of free sugars to <5% of total energy intake for adults [30]. Dietary Guidelines for Americans (2020–2025) recommend that a healthy dietary pattern limits added sugars to less than 10 percent of calories per day [31]. According to the dietary guidelines for Chinese residents, it recommended to limit the intake of added sugars to no more than 50 g/day, preferably under 25 g/day [32]. In this study, free sugars intake of 5–20 g/day was positively associated with MetS among adults. A modeling study has found that a reduction in free sugars added to sugar-sweetened beverages was predicted to reduce the prevalence of overweight, obesity, and type 2 diabetes [33].

Many current studies indicate that both added and free sugars are recommended to be consumed in moderation. Compared with the consumption level of western countries, the consumption of free sugars—particularly in the form of SSBs—in China is still at a relatively low level. Thus, the risk of chronic non-communicable diseases is relatively low with free sugars intake at lower levels in China [26,34]. Nevertheless, with the development of the economy and changes of lifestyle, the eating behavior and diet is also changing in China. Traditional dietary patterns in China are shifting to western dietary patterns with an increase in packaged foods consumption such as SSBs [35]. Mullee et al. showed that higher all-cause mortality was found in participants who consumed ≥2 glasses per day of SSBs compared to consumers of <1 glass per month [36]. A recent study showed that the number of deaths was 46,633 in 2019 attributed to high SSBs intake in China, which is about double the number in 1990 [37]. Considering the growing trend of sugary food and SSBs consumption and the increasing prevalence of MetS, it is necessary to pay more attention to the problem of excessive intake of free sugars in Chinese adults.

The strengths of our study include using a large participant size and longer follow-up data from CHNS. Moreover, we adopted a multivariable Cox of proportional hazards model to classify the association between free sugars intake and the risk of MetS. However, our study still had several limitations that deserve discussion. Firstly, it may be possible for recall bias to occur because of the dietary intake assessment using three 24 h recalls. Secondly, the possibility of residual confounding cannot be excluded completely on the account of the nature of observational studies. Thirdly, we used the sugar content of sugary foods that are commonly available on the market. There may be differences of sugar content among different batches of sugary foods.

## 5. Conclusions

This present prospective study provides evidence that free sugars intake of 5–20 g/day is positively correlated with MetS in Chinese adults, especially among women. Therefore, the mechanism of free sugars with MetS and its single component between sexes is worth detailed study in the future. We suggest that attention should be paid to the intake of free sugars and comprehensive measures such as the improvements in the processing of sugary foods, and sugar composition should be included on food nutrition labels to control the intake of free sugars in China.

## Figures and Tables

**Table 1:** Basic characteristics of overall participants according to different levels of free sugars intake, CHNS.

	Overall Participants				
	<5 g/Day	5–20 g/day	>20 g/day	χ^2^	*p*
Number of subjects	9056 (75.2)	2645 (22.0)	347(2.9)		
Age					
18–44	4872 (76.5)	1326 (20.8)	172 (2.7)	12.61	<0.001
≥45	4184 (73.7)	1319 (23.2)	175 (3.1)		
Education level					
Junior high school or below	6333 (81.4)	1322 (17.0)	128 (1.6)	476.28	<0.001
Senior high school or above	2723 (63.9)	1323 (31.0)	219 (5.1)		
Place of residence					
Urban area	2573 (60.1)	1451 (33.9)	260 (6.1)	866.51	<0.001
Rural area	6483 (83.5)	1194 (15.4)	87 (1.1)		
Region of residence					
Northern region	3741 (74.8)	1127 (22.5)	131 (2.6)	3.48	0.175
Southern region	5315 (75.4)	1518 (21.5)	216 (3.1)		
Individual annual income					
Low	3575 (89.1)	417 (10.4)	20 (0.5)	1199.90	<0.001
Medium	3191 (79.3)	763 (19.0)	72 (1.8)		
High	2290 (57.1)	1465 (36.5)	255 (6.4)		
Drinking history					
Yes	4762 (75.8)	1363 (21.7)	160 (2.6)	6.16	0.046
No	4294 (74.5)	1282 (22.3)	187 (3.2)		
Smoking history					
Yes	3930 (77.6)	1018 (20.1)	118 (2.3)	29.73	<0.001
No	5126 (73.4)	1627 (23.3)	229 (3.3)		
Physical activity					
Low	2767 (69.0)	1070 (26.7)	175 (4.4)	254.37	<0.001
Medium	2942 (73.1)	964 (24.0)	119 (3.0)		
High	3347 (83.5)	611 (15.2)	53 (1.3)		
Intake of total energy					
Low	3015 (75.2)	910 (22.7)	87 (2.2)	27.68	<0.001
Medium	3036 (75.4)	888 (22.1)	101 (2.5)		
High	3005 (74.9)	847 (21.1)	159 (4.0)		

**Table 2:** Basic characteristics of men according to different levels of free sugars intake, CHNS.

	Men				
	<5 g/Day	5–20 g/day	>20 g/day	χ^2^	*p*
Number of subjects	4407 (36.6)	1236 (10.3)	169 (1.4)		
Age					
18–44	2386 (37.5)	619 (9.7)	85 (1.3)	6.97	<0.05
≥45	2021 (35.6)	617 (10.9)	84 (1.5)		
Education level					
Junior high school or below	2832 (36.4)	567 (7.3)	66 (0.9)	166.14	<0.001
Senior high school or above	1575 (36.9)	669 (15.7)	103 (2.4)		
Place of residence					
Urban area	1240 (28.9)	644 (15.0)	126 (2.9)	368.01	<0.001
Rural area	3167 (40.8)	592 (7.6)	43 (0.6)		
Region of residence					
Northern region	1801 (36.0)	533 (10.7)	67 (1.3)	2.23	0.329
Southern region	2606 (37.0)	703 (10.0)	102 (1.5)		
Individual annual income					
Low	1740 (43.4)	205 (5.1)	10 (0.3)	520.94	<0.001
Medium	1543 (38.3)	365 (9.1)	38 (0.9)		
High	1124 (28.0)	666 (16.6)	121 (3.0)		
Drinking history					
Yes	3719 (59.2)	990 (15.8)	122 (1.9)	27.50	<0.001
No	688 (11.9)	246 (4.3)	47 (0.8)		
Smoking history					
Yes	3599 (71.0)	939 (18.5)	108 (2.1)	47.42	<0.001
No	808 (11.6)	297 (4.3)	61 (0.9)		
Physical activity					
Low	1357 (33.8)	513 (12.8)	82 (2.0)	104.11	<0.001
Medium	1417 (35.2)	422 (10.5)	56 (1.4)		
High	1633 (40.7)	301 (7.5)	31 (0.8)		
Intake of total energy					
Low	745 (18.6)	225 (5.6)	17 (0.4)	14.71	<0.05
Medium	1388 (34.5)	400 (9.9)	43 (1.1)		
High	2274 (56.7)	611 (15.2)	109 (2.7)		

**Table 3:** Basic characteristics of women according to different levels of free sugars intake, CHNS.

	Women				
	<5 g/Day	5–20 g/day	>20 g/day	χ^2^	*p*
Number of subjects	4649 (38.6)	1409 (11.7)	178 (1.5)		
Age					
18–44	2486 (39.0)	707 (11.1)	87 (1.4)	5.73	<0.057
≥45	2163 (38.1)	702 (12.4)	91 (1.6)		
Education level					
Junior high school or below	3501 (45.0)	755 (9.7)	62 (0.8)	341.45	<0.001
Senior high school or above	1148 (26.9)	654 (15.3)	116 (2.7)		
Place of residence					
Urban area	1333 (31.1)	807 (18.8)	134 (3.1)	500.95	<0.001
Rural area	3316 (42.7)	602 (7.8)	44 (0.6)		
Region of residence					
Northern region	1940 (38.8)	594 (11.9)	64 (1.3)	2.54	0.281
Southern region	2709 (38.4)	815 (11.6)	114 (1.6)		
Individual annual income					
Low	1835 (45.7)	212 (8.3)	10 (0.3)	680.75	<0.001
Medium	1648 (40.9)	398 (9.9)	34 (0.8)		
High	1166 (29.1)	799 (19.9)	134 (3.3)		
Drinking history					
Yes	1043 (16.6)	373 (5.9)	38 (0.6)	10.26	<0.05
No	3606 (62.6)	1036 (18.0)	140 (2.4)		
Smoking history					
Yes	331 (6.5)	79 (1.6)	10 (0.2)	4.30	0.116
No	4318 (61.8)	1330 (19.1)	168 (2.4)		
Physical activity					
Low	1410 (35.1)	557 (13.9)	93 (2.3)	154.63	<0.001
Medium	1525 (37.9)	542 (13.5)	63 (1.6)		
High	1714 (42.7)	310 (7.7)	22 (0.6)		
Intake of total energy					
Low	2270 (56.6)	685 (17.1)	70 (1.7)	20.04	<0.001
Medium	1648 (40.9)	488 (12.0)	58 (1.4)		
High	731 (18.2)	236 (5.9)	50 (1.3)		

**Table 4:** Association between free sugars intake and the risk of MetS.

	<5 g/Day	5–20 g/Day	>20 g/Day
Overall Participants			
Model 1	1.00 (Reference)	1.084 (0.999, 1.175)	0.991 (0.783, 1.253)
Model 2	1.00 (Reference)	1.095 (1.009, 1.188) *	0.966 (0.763, 1.224)
Model 3	1.00 (Reference)	1.094 (1.009, 1.186) *	1.011 (0.800, 1.277)
Men			
Model 1	1.00 (Reference)	1.058 (0.940, 1.192)	1.039 (0.757, 1.428)
Model 2	1.00 (Reference)	1.078 (0.958, 1.212)	1.037 (0.756, 1.422)
Model 3	1.00 (Reference)	1.077 (0.957, 1.213)	1.073 (0.782, 1.473)
Women			
Model 1	1.00 (Reference)	1.111 (0.995, 1.242)	0.922 (0.651, 1.308)
Model 2	1.00 (Reference)	1.122 (1.003, 1.255) *	0.895 (0.630, 1.272)
Model 3	1.00 (Reference)	1.105 (0.990, 1.232)	0.918 (0.650, 1.298)

Model 1 adjusted for gender, age, educational level, place of residence, region of residence, individual annual income, drinking history, and smoking history. Model 2 was further adjusted for physical activity based on Model 1. Model 3 was further adjusted for intakes of total energy based on Model 2. * *p* < 0.05.

## Data Availability

Data sharing is not applicable to this article.
